# Transcriptomic changes in embryonic blood of subsequent generations following *in ovo* stimulation with nutriepigenetic factors

**DOI:** 10.1186/s12864-026-12983-6

**Published:** 2026-06-12

**Authors:** Mariam Ibrahim, Katarzyna Stadnicka, Marek Bednarczyk, Ewa Grochowska

**Affiliations:** 1 Faculty of Health Sciences, Collegium Medicum, Nicolaus Copernicus University, Łukasiewicza 1, Bydgoszcz, 85-821 Poland; 2 Department of Animal Biotechnology and Genetics, Bydgoszcz University of Science and Technology, Mazowiecka 28, Bydgoszcz, 85-084 Poland; 3 Institute of Advanced Studies, FemLife-OMICS, Nicolaus Copernicus University, Wieńska 4, Toruń, 87-100 Poland

## Abstract

**Background:**

Epigenetic modifications regulate gene expression and are influenced by environmental factors, shaping phenotypic and clinical outcomes. These changes have the potential to persist across generations, although their stability may vary between tissues. This study aims to observe, using an *in ovo* model, the effects of single (F1) and repeated (F1-F3) prenatal stimulation with potential epigenetic factors on the transcriptome of embryonic blood in subsequent generations.

**Method:**

Using an *in ovo* model across three generations of Green-legged Partridgelike chickens (with additional F4 assessment), synbiotic PoultryStar^®^ (PS) and choline were injected on day 12 of incubation. F1 embryos were assigned to control (0.9% NaCl), synbiotic (SYN, 2 mg PS), or synbiotic + choline (SYNCH, 2 mg PS + 0.25 mg choline) groups. In F2 and F3, SYN and SYNCH were split into two subgroups each: (A) injected only once in F1 embryos (SYNs and SYNCHs); and (B) repeatedly injected in every successive generation (SYNr and SYNCHr). Embryonic blood was collected from the dorsal aorta at HH stages 14–16, and RNA sequencing was performed on male samples.

**Results:**

Transcriptomic changes in F3 embryonic blood were found in treatment groups vs. control after single (F1) and repeated (F1-F3) *in ovo* administration of synbiotic alone and synbiotic combined with choline. Gene expression differences detected in F3 embryonic blood in a single ancestral *in ovo* exposure lineages largely diminished in F4, suggesting that effects of these treatments may attenuate with generational distance. Repeated injections in SYNr and SYNCHr groups did not produce cumulative effects in F3 and F4 generations. Gene set enrichment analysis indicated that the most affected functional categories involved metabolism, detoxification, cytoskeletal organization, and protein regulation.

**Conclusions:**

These findings highlight that targeted prenatal interventions can induce transcriptomic modifications, which may be detectable in the subsequent generations (F3), though their persistence through the further generations may be limited. Embryonic blood may reflect a potential epigenetic footprint rather than a stably inherited signal. The tissue-specific and time-sensitive nature of the transcriptomic effects confirms the importance of analysis across different tissues, and across generations.

**Supplementary Information:**

The online version contains supplementary material available at 10.1186/s12864-026-12983-6.

## Background

Epigenetic mechanisms, including DNA methylation, histone modification, and regulation by non-coding RNAs, are crucial in linking the environmental impact to gene expression [1]. These mechanisms are highly sensitive to environmental inputs, especially during critical developmental windows such as prenatal and early postnatal periods [2].

In some cases, the consequences of epigenetic modulation can be transferred to the germline and passed on beyond the directly exposed individual, influencing the phenotype of subsequent generations [3, 4]. However, such transgenerational effects remain difficult to conclusively demonstrate, particularly in vertebrates, where inherited molecular changes can be difficult to distinguish from direct environmental influences [4]. Transmission of epigenetic information may be dynamic, and may involve non-linear, time-dependent changes that challenge a simplistic on–off model, with effects that can gradually “wash in” or “wash out” across generations in response to environmental stressors [5]. Understanding these dynamics requires sensitive, quantitative methods and a focus on how epigenetic patterns evolve over time [5].

*In ovo* models provide unique advantages for studying inter- and transgenerational effects of epigenetic factors without the confounding effects of in utero influences, due to their external embryonic development and accessibility of egg content for experimental manipulation [6, 7]. Of particular interest is the chicken embryonic blood, which in early stages contains both somatic blood cells and circulating primordial germ cells (PGCs) before they migrate to the gonads [8]. As such, embryonic blood may offer a valuable snapshot of systemic gene regulation that may include components relevant to both somatic and germline lineages. The epigenome of chicken erythrocytes responds to both internal factors, such as metabolism, and external influences like the environment, affecting chromatin structure and gene expression [9]. With a compact genome that preserves many conserved syntenic blocks with the human genome, chicken red blood cells may serve as an effective model for exploring how environmental conditions shape the epigenome and for drawing preliminary parallels to human health [9].

Despite increasing interest, few studies have explored transcriptomic changes in transgenerational transmission in avian model, and even fewer have examined gene expression in embryonic blood at later generations such as F3 and F4 [10–12]. The objective of this study was to examine changes in gene expression patterns in the embryonic blood of F3 and F4 chicken generations following single ancestral (F1) or repeated (F1-F3) *in ovo* exposure to potential epigenetic modulators, choline and synbiotic (PoultryStar^®^, further referred to as PS).

## Methods

### Ethical consideration

The study adhered to the ethical standards outlined in Directive 2010/63/EU and Regulation (EU) 2019/1010. This research has been reported following the ARRIVE guidelines [13] (https://arriveguidelines.org*).* Animal care protocols, including daily welfare monitoring by trained personnel and a veterinarian, were consistent with those applied in our previously published results of this study [14, 15]. The birds were raised under standard environmental conditions in a poultry farm.

### Birds and experimental design

This study was conducted using Green-legged Partridgelike chickens, a native Polish slow-growing breed known for its adaptability and resilience. These birds are well-suited to various environmental conditions due to their minimal nutritional needs, strong immunity, and natural resistance to harsh climates [16, 17]. Additionally, they exhibit strong maternal traits. Unlike commercial poultry breeds, this breed has undergone minimal selective breeding, preserving greater genetic diversity, making it a valuable model for transgenerational research [16]. Green-legged Partridgelike chickens are outbred lines, which may potentially exhibit greater susceptibility to transmission of epigenetic modifications to the subsequent generations than inbred lines, making them a good model for studying transgenerational effects [10]. This phenomenon has been observed in rodents [18].

**Fig. 1:**
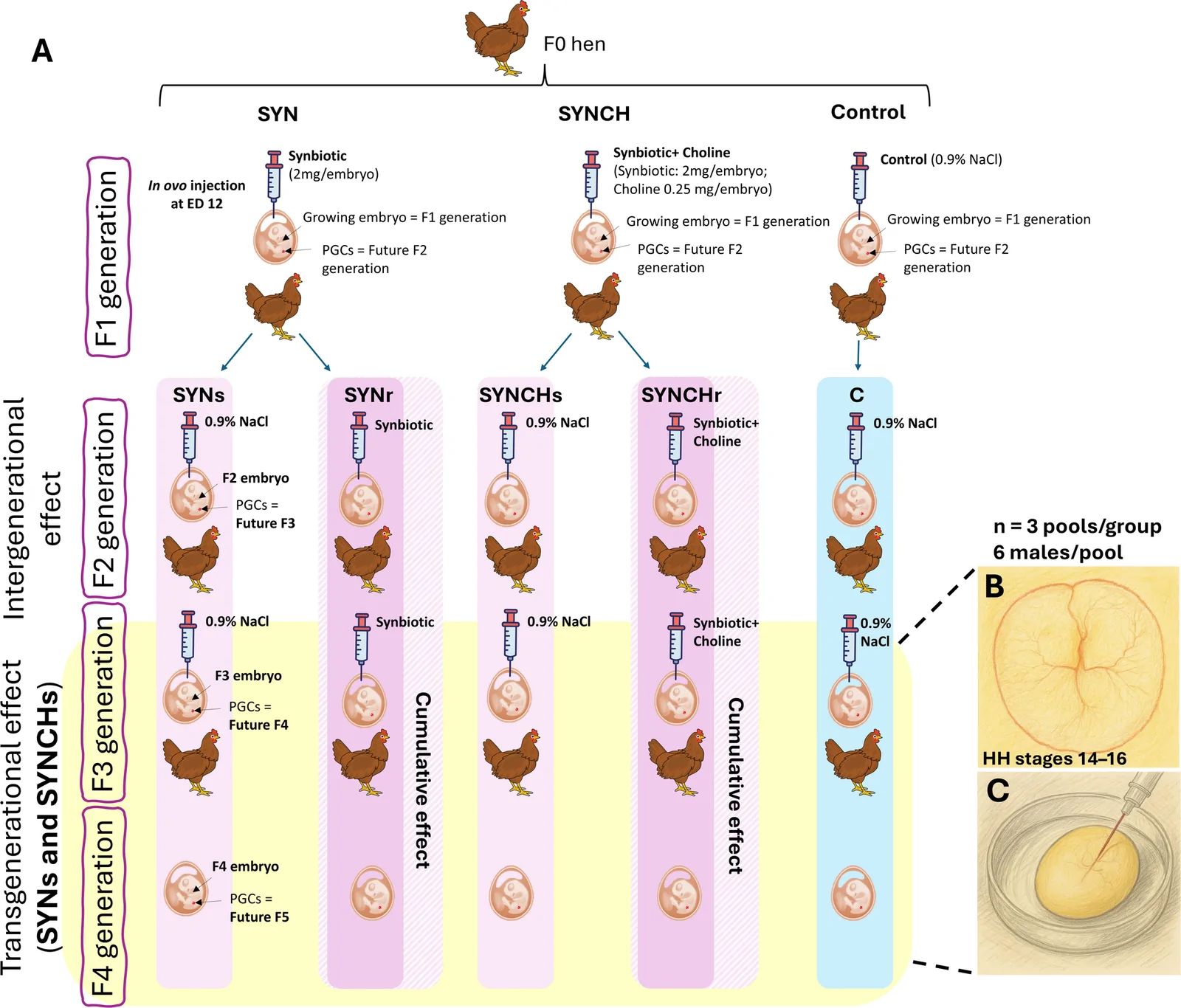
Experimental design of the study. (**A**) The study was conducted over three generations with additional assesment of F4 embryos. F1 embryos were injected at embryonic day (ED) 12 with either a synbiotic, a combination of synbiotic and choline, or 0.9% physiological saline (NaCl). In subsequent generations, fertilized eggs from each treatment group were divided into two subgroups: one that continued without further injections of synbiotic or a combination of synbiotic and choline (SYNs and SYNCHs), and another that received repeated injections of synbiotic or a combination of synbiotic and choline at ED12 in F2 and F3 generations (SYNr and SYNCHr). (**B**) Fertilized eggs from the F2 and F3 generations were incubated for 2.5 days, reaching Hamburger-Hamilton (HH) stages 14–16. (**C**) Embryonic blood was collected from the dorsal aorta using a fine glass microcapillary pipette. For generations F3 and F4, embryonic blood was collected from 6 male embryos and pooled to form one biological replicate, yielding *n* = 3 independent pools per experimental group for RNA sequencing

Figure 1 presents the study design, which was detailed in our previous papers [14, 15]. The experimental design, including injection timing, dosage, and breeding strategy, was consistent with our prior work exploring cecal and gonadal transcriptomes as well as immune system phenotype [14, 15, 19], ensuring cross-tissue comparability of transgenerational responses to *in ovo* stimulation. Briefly, the experiment spanned three generations (F1–F3), starting from fertilized eggs (F1 embryos) of Green-legged Partridgelike hens from the F0 generation. These eggs were purchased from Zofia i Gracjan Skórniccy Hodowla Kur Zielononóżek (Duszniki, Poland). In this study, F4 embryos were also included. On embryonic day 12, eggs with viable F1 embryos were injected manually into the air cell with one of the following treatments (3 groups): (1) Synbiotic group (SYN) – received an injection of 2 mg/embryo of synbiotic PoultryStar^®^ sol^US^ (DSM-Firmenich, Kaiseraugst, Switzerland) suspended in 0.2 mL of 0.9% NaCl; (2) Synbiotic and choline group (SYNCH) – received 2 mg/embryo of synbiotic PS combined with 0.25 mg/embryo of choline (Sigma Alrich, Sain Louis, MA, USA, cat. no. C7527), suspended in 0.2 mL of 0.9% NaCl; (3) Control group (C) – received an injection of 0.2 mL of 0.9% NaCl. From the F2 generation onward, the treatment groups were divided into four subgroups: (1) SYNs – single synbiotic PS injection applied only in F1 embryos; (2) SYNCHs – single synbiotic PS + choline injection applied only in F1 embryos; (3) SYNr – repeated synbiotic PS injections in F2 and F3 generations; (4) SYNCHr–repeated synbiotic + choline injections in F2 and F3 generations, in addition to a control group (0.9% NaCl). The selection of the choline source, its dosage, and the combined synbiotic and choline dosages was guided by findings from two experiments described in our previous study [14]. The *in ovo* injection protocol was based on the optimized method of Bednarczyk et al. [20, 21]. Housing and feeding protocols for chickens were described previously [14, 15].

### Embryonic blood isolation

Eggs (*n* = 20–28 per group, Supplementary file S1) were incubated under standard conditions (37.5 °C, 55% relative humidity, turned every 2 h) for 2.5 days, until the embryos reached HH-stage 14–16. Embryonic blood containing cPGCs was extracted from the dorsal aorta of individual F3 and F4 embryos under a stereomicroscope (Supplementary video). A fine glass microcapillary pipette (inner diameter: 30 μm, outer diameter: 40 μm) connected to a mouth pipette was used for collection (Sigma Alrich, Saint Louis, MA, USA, cat. no. A5177).

Blood samples from every embryo were individually transferred into Eppendorf tubes containing RNALater (ThermoFisher, Waltham, MA, USA, cat. no. AM7021) and stored at 4 °C until later usage. After determining the sex of the embryos (methodology is described in the next subchapter), samples were pooled into male and female groups, with only male samples being used in this study. Pooling was done in such a way that: (i) each pool consisted of blood from 6 male embryos (*n* = 6 male embryos per pool); (ii) pool sizes were consistent across groups and generations; (iii) pools were constructed from embryos originating from different incubation batches (i.e., different clutches) to represent independent biological replicates; and (iv) each pool was treated as one independent biological replicate in further analyses (RNA-sequencing and DESeq2; *n* = 3 per group). Full details of pool composition, incubation outcomes, and library identifiers for both the F3 and F4 generations are provided in Supplementary file S1. The collected blood was separated through centrifugation in RNase-free water at 10,000 × g for three minutes. RNA extraction was then performed using the GeneMATRIX Universal RNA Purification Kit (EURx, Gdańsk, Poland, cat. no. E3598) following the manufacturer’s protocol.

RNA integrity was assessed using an Agilent Bioanalyzer 2100 system (Agilent Technologies, Santa Clara, CA, USA) with an RNA Nano 6000 Assay Kit. Additionally, RNA quality was checked by electrophoresis on a 1% agarose gel. All extracted RNA samples met the required quality standards, achieving an RNA integrity number (RIN) of 9.0 or higher, confirming their suitability for downstream applications.

### Sex determination

DNA was extracted from each embryo, corresponding to its respective isolated blood sample, using the QIAamp Fast DNA Tissue Kit (Qiagen, Hilden, Germany, Cat. No. 51404), following the manufacturer’s instructions. Sex determination was carried out as previously described [22]. Briefly, embryo samples were homogenized by vortexing in lysis buffer for 30 s, followed by incubation in a thermomixer (TS-100 C, Biosan, Riga, Latvia) at 56 °C with a shaking speed of 1000 rpm for 5 min. Sex determination of the embryos was performed using two pairs of primers: one specific to the female *XhoI* W-repeat sequence (5′-*CCCAAATATAACACGCTTCACT*-3′ and 5′-*GAAATGAATTATTTTCTGGCGAC*-3′), and another targeting the 18 S ribosomal gene (5′-*AGCTCTTTCTCGATTCCGTG*-3′ and 3′-*GGGTAGACACAAGCTGAGCC*-3′), as described by Clinton et al. [23].

The PCR-amplified products were separated using electrophoresis on a 2% agarose gel stained with MIDORI Green Advance (NIPPON Genetics, Düren, Germany, cat. no. MG04). The gel was run at 110 V for 35 min, and DNA bands were visualized and photographed using the G: Box Chemi XR5 imaging system (SYNGENE, Cambridge, UK). In female samples, two distinct bands are observed: one corresponding to the female-specific *XhoI* W-repeat sequence (415 base pairs) and the other to the 18 S ribosomal gene (256 base pairs), which serves as an internal PCR control. In contrast, male embryos exhibit only the 18 S ribosomal gene band. Only male samples were included in this study.

### RNA-sequencing and analysis

A total of 30 RNA-seq libraries were generated (15 libraries per generation (F3 and F4), with 3 libraries per treatment and control group) using the Novogene NGS Stranded RNA Library Prep Set (PT044, Novogene, Cambridge, UK). Sequencing was performed on the Illumina NovaSeq 6000 platform (Novogene, Cambridge, UK) at a depth of 20 million forward and 20 million reverse reads per sample, utilizing a 150 paired-end sequencing kit. Quality control of the raw sequencing data was assessed using FastQC v0.12.1 [24]. Adapter sequences and low-quality reads were removed using fastp v0.23.4 to obtain high-quality clean data for further analysis [25]. The Q20, Q30, and GC content of the processed reads were then evaluated. Following preprocessing, paired-end reads were aligned to the chicken genome (bGalGal1.mat.broiler.GRCg7b) using STAR 2.7.11b [26]. Differential gene expression analysis was performed using DESeq2 v1.42.0 [27] in RStudio (2025.5.0.496) [28]. Raw counts were normalized within the DESeq2 package, and differentially expressed genes (DEGs) were identified based on an adjusted p-value (≤ 0.05) and absolute log2 fold change threshold of 0.585. Over-representation analysis (ORA) for Gene Ontology (GO) and Kyoto Encyclopedia of Genes and Genomes (KEGG, https://www.kanehisa.jp/ [29]) pathways was performed using clusterProfiler [30]. Multiple testing correction was applied using the Benjamini-Hochberg (BH) method (pAdjustMethod = “BH”). Significance thresholds were set at a p-value ≤ 0.05 and a false discovery rate (q-value) ≤ 0.10. Only terms and pathways with at least three implicated DEGs were considered. Gene set enrichment analysis (GSEA) for KEGG pathways and GO terms were also performed [31] using clusterProfiler package (v.4.10.1) in RStudio (2025.5.0.496) [28]. For both KEGG pathways and GO terms enrichment, genes were ranked by the DESeq2 Wald test statistic, and GSEA was implemented using the fgsea algorithm via the clusterProfiler package (v4.10.1). GO term annotations were sourced from the org.Gg.eg.db Bioconductor annotation package (v3.18.0), and enrichment was assessed separately for Biological Process (BP), Cellular Component (CC), and Molecular Function (MF) ontologies. KEGG pathway annotations for Gallus gallus were retrieved from the KEGG database using the KEGGREST package (v1.42.0), with Entrez IDs converted to gene symbols and formatted as a GMT file (accessed June 2025). For all GSEA analyses, gene-set size was restricted to a minimum of 3 and a maximum of 800 genes. Multiple testing correction was applied using the BH method, with significance defined as adjusted p-value < 0.05. Results were further filtered by an absolute normalized enrichment score |NES| ≥ 1.5 and a minimum of three core enrichment genes. Principal component analysis (PCA) was performed on variance-stabilizing transformation (VST)-normalized counts obtained via DESeq2 (v1.42.0), and visualized as pairwise comparisons between the control group and each treatment subgroup. The function and expression information of the common genes were retrieved from UniProt (Universal Protein Resource; https://www.uniprot.org/uniprotkb) [32] and NCBI (National Center for Biotechnology Information; https://www.ncbi.nlm.nih.gov/gene/). Direct cross-generational comparisons of treatment effects were not performed as the primary analysis, as each generation was compared to its own concurrent control to account for any generational drift in baseline expression. The pattern of DEG numbers across generations was instead used to infer persistence or attenuation of effects.

### Validation of sequencing data by quantitative reverse transcription-polymerase chain reaction (RT-qPCR)

To validate the RNA sequencing data, five upregulated and five downregulated DEGs involved in various KEGG pathways were selected for RT-qPCR analysis, with three biological replicates for each sample. Complementary DNA (cDNA) was synthesized using the smART First Strand cDNA Synthesis Kit (EURx, Gdańsk, Poland, Cat. No. E0804), and quantitative real-time PCR (qPCR) was performed with primers listed in Supplementary file S2, designed using Primer-BLAST [33]. Each reaction was conducted in a 20-µL volume containing 50 ng of cDNA, 0.25U uracil-N-glycosylase (UNG), and 15 pmol of each forward and reverse primer, with 1× SG qPCR Master Mix (Eurx, Gdańsk, Poland, Cat. No. E0401).

The qPCR thermocycling conditions included an initial UNG pre-treatment at 50 °C for 2 min, followed by 1 cycle of denaturation at 95 °C for 10 min, and 40 amplification cycles at 94 °C for 15 s, 60 °C for 30 s, and 72 °C for 30 s. To ensure the specificity of the amplicons, a melting curve analysis was performed with the following conditions: 95 °C for 5 s, 70 °C for 5 s, and a gradual increase to 95 °C at a ramp rate of 0.5 °C/cycle. Amplifications were conducted using the CFX Opus 96 real-time PCR system (BIO-RAD, CA, USA). Gene expression levels were analyzed using the Pfaffl (or standard curve) method [34]. Pearson’s correlation analysis was performed using RStudio (2025.5.0.496) [28] to evaluate the association between log2 fold changes obtained from RNA-seq and qPCR experiments.

## Results

### RNA sequencing and alignment summary statistics

A total of 30 embryonic blood samples were sequenced using the Illumina NovaSeq 6000 sequencing platform. After removing adapter sequences and low-quality reads, the average number of clean reads obtained in the experimental groups ranged from 16,846,058 to 30,562,766 in F3, and from 17,557,189 to 34,060,498 in F4 (Supplementary file S3). Across all samples, the quality metrics were high, with 99.99% of bases achieving Q20 and at least 97.9% reaching Q30. The GC content ranged from 48% to 54%. Additionally, 84% to 89.85% of clean reads uniquely mapped to the chicken reference genome (bGalGal1.mat.broiler. GRCg7b; NCBI RefSeq assembly: GCF_016699485.2, annotation release 106) (Supplementary file S3).

PCA of VST-normalised RNA-seq data from embryonic blood was performed pairwise between the control and each treatment subgroup across F3 and F4 generations (Supplementary file 4, Figure S1). In F3, the greatest PC1 separation was observed in C vs. SYNs (36.4%) and C vs. SYNr (35.8%), followed by C vs. SYNCHs (31.4%) and C vs. SYNCHr (29.6%). Within-group variance was present across all comparisons, with individual replicates displaced from their group clusters in several panels (e.g. SYNs1F3, SYNr1F3, SYNCHs3F3). In F4, PC1 values were higher in C vs. SYNs (56%) and C vs. SYNCHs (54.2%), driven by individual outlier samples rather than coherent group separation, while C vs. SYNr (34.6%) and C vs. SYNCHr (48.1%) showed more distributed scatter. Overall, within-group variance was greater in F4 than in F3 across all comparisons.

### Identification of differentially expressed genes (DEGs)

**Fig. 2:**
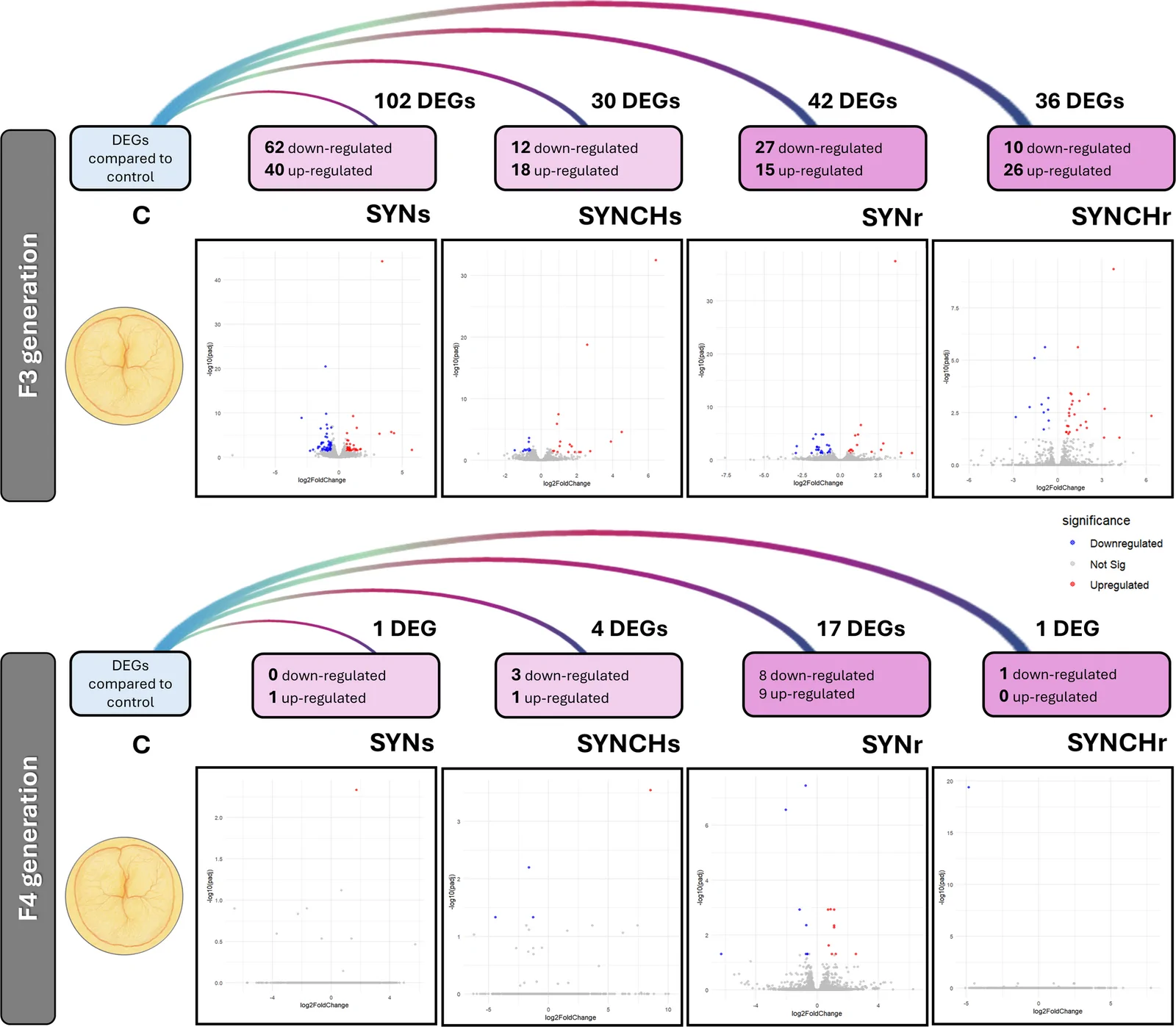
Diagram showing the number of differentially expressed genes (DEGs) identified by comparing the experimental groups with the control group in F3 and F4 generations (*n* = 3 samples per group per generation). C: control group; SYNs: group that received a single injection of synbiotic PoultryStar^®^ (PS) in F1 embryos; SYNr: group that received repeated synbiotic PS injections (F1-F3); SYNCHs: group that received a single injection of synbiotic PS combined with choline in F1 embryos; and SYNCHr: group that received repeated injections of synbiotic PS combined with choline (F1-F3)

Following mapping and quality control, a total of 25,470 expressed genes were retained for differential expression analysis. Figure 2 presents the number of differentially expressed genes (DEGs, adjusted p-value ≤ 0.05; absolute log2 fold change (|log2FC|) cutoff of 0.585) detected in comparisons between the experimental groups and the control group in F3 and F4 embryos.

In the F3 generation, the SYNs group exhibited the highest number of DEGs (*n* = 102), while the SYNCHs group showed 30 DEGs. Groups subjected to repeated stimulation showed 42 DEGs in SYNr and 36 in SYNCHr. In the F4 generation, the number of DEGs declined markedly in all groups. Only one DEG was identified in the SYNs group, while the SYNCHs group had four. The SYNr and SYNCHr groups showed 17 and one DEG, respectively. The complete lists of DEGs for all comparisons are provided in Supplementary file S5.

When comparing gene expression between F3 treatment groups—one receiving a single F1 injection versus another receiving injections across multiple successive generations (F1-F3), we found eleven shared genes between SYNs and SYNr (*HIST1H4D*, *LOC124417784*, *DSE*, *CYP3A4*, *COX14*, *LOC112531967*, *LOC112532140*, *J6367_mgt12*,* SERF2*, *PHKA1* and *MITD1*), and five shared between SYNCHs and SYNCHr (*LOC124417784*, *TMEM151B*, *BMP5*, *CYP3A4* and *SPECC1*). No overlapping DEGs were detected across the experimental groups in F4 embryos. Table 1 summarizes the putative functions of the common DEGs identified. Cross-generational comparisons of corresponding groups in subsequent generations revealed one common gene shared between SYNs group in F3 and SYNs group in F4 (*HBBA*), and no overlapped DEGs between SYNCHs in F3 and that in F4. The SYNr groups showed one overlapping gene between F3 and F4 (*BG8*), whereas no shared DEGs were found in the SYNCHr groups across generations. Interestingly, the expression of the aforementioned genes (*HBBA and BG8)* was downregulated in F3 embryos while upregulated in F4 embryos. Table 2 outlines the potential functions of the two shared DEGs observed across comparable groups in the F3 and F4 generations.

**Table 1:** Shared differentially expressed genes between SYNs and SYNr groups, and between SYNCHs and SYNCHr groups in F3

Comparison	Gene symbol	Description	Expression		Function
SYNs-SYNr	HIST1H4D	Histone Cluster 1 H4 Family Member D		↑	Core component of nucleosome; plays a central role in transcription regulation, DNA repair, DNA replication, and chromosomal stability.
	LOC124417784	Uncharacterized LOC124417784		↑	Function not characterized.
	DSE	Dermatan Sulfate Epimerase		↑	Converts D-glucuronic acid to L-iduronic acid residues; important in dermatan sulfate biosynthesis.
	CYP3A4	Cytochrome P450 Family 3 Subfamily A Member 4		↑	Involved in metabolism of sterols, steroid hormones, retinoids, fatty acids, and xenobiotics.
	COX14	Cytochrome C Oxidase Assembly Factor COX14		↓	Regulates cytochrome c oxidase assembly; essential for mitochondrial function.
	LOC112531967	Uncharacterized LOC112531967		↓	Potentially associated with viral processes.
	LOC112532140	Uncharacterized LOC112532140		↓	Potentially involved in immune responses to viral infections.
	J6367_mgt12	Uncharacterized Gene J6367_mgt12		↓	Related to tRNA-Ser.
	SERF2	Small EDRK-rich factor 2		↓	Positive regulator of amyloid protein aggregation and proteotoxicity.
	PHKA1	Phosphorylase b kinase regulatory subunit alpha 1		↑	Phosphorylase b kinase catalyzes the phosphorylation of serine in certain substrates. The alpha chain may bind calmodulin.
	MITD1	Microtubule-Interacting and Trafficking Domain Protein 1		↓	Required for efficient abscission at the end of cytokinesis, together with components of the ESCRT-III complex.
SYNCHs-SYNCHr	LOC124417784	Uncharacterized LOC124417784		↑	Function not characterized.
	TMEM151B	Transmembrane Protein 151B		↑	Encodes a protein with two transmembrane domains; function not well-defined.
	BMP5	Bone Morphogenetic Protein 5		↑	Growth factor from the TGF-beta superfamily; involved in cartilage and bone formation, and neurogenesis.
	CYP3A4	Cytochrome P450 Family 3 Subfamily A Member 4		↑	Involved in metabolism of sterols, steroid hormones, retinoids, fatty acids, and xenobiotics.
	SPECC1	Sperm Antigen with Calponin Homology and Coiled-Coil Domains 1		↑	Involved in cytokinesis and spindle organization; may play a role in actin cytoskeleton organization and microtubule stabilization.

Gene functions are retrieved from the UniProt (https://www.uniprot.org/uniprotkb/) and NCBI (https://www.ncbi.nlm.nih.gov/gene/)

SYNs: group that received a single injection of synbiotic PoultryStar^®^ (PS) in F1 embryos; SYNr: group that received repeated synbiotic PS injections (F1-F3); SYNCHs: group that received a single injection of synbiotic PS combined with choline in F1 embryos; and SYNCHr: group that received repeated injections of synbiotic PS and choline (F1-F3)

**Table 2:** Common differentially expressed genes between analogous SYNs and SYNr Groups in F3 and F4

Comparison	Gene symbol	Description	Expression	Function
SYNsF3-SYNsF4	HBBA	Hemoglobin Beta, Subunit A	↓ in F3 ↑ in F4	Involved in oxygen transport from the lung to the various peripheral tissues
SYNrF3-SYNrF4	BG8	BG Gene 8 (Major Histocompatibility Complex Class IV)	↓ in F3 ↑ in F4	Involved in the immune system, particularly in antigen presentation and immune response modulation

Gene functions are retrieved from the UniProt (https://www.uniprot.org/uniprotkb/)

SYNs: group that received a single injection of synbiotic PoultryStar^®^ (PS) in F1 embryos; SYNr: group that received repeated synbiotic PS injections (F1-F3)

### Functional clustering of DEGs

Gene Ontology (GO) enrichment analysis was performed on DEGs from the embryonic blood of F3 and F4 embryos to investigate the functional significance of transcriptional changes. Enriched GO terms were classified into three categories: biological process (BP), cellular component (CC), and molecular function (MF). Over-representation analysis (ORA) using the KEGG and GO databases revealed no significant enrichment in F3. In F4 embryos, the limited number of identified DEGs was insufficient to support robust functional annotation.

We further performed gene set enrichment analysis (GSEA) for GO terms and KEGG pathways to investigate the systemic regulation of the entire gene sets in embryonic blood in F3 and F4 generations (Supplemantary file 4, Figures S2, S3 and S4). Supplementary file 4 (Table S1) shows the number of enriched pathways and GO terms by GSEA in each generation. The full lists of enriched GO terms and KEGG pathways by GSEA are shown in Supplementary files S6 and S7, respectively.

In F3 embryos, SYNs and SYNr are predicted to activate protein-related and metabolic processes (Supplementary file 4, Figure S2). By F4, SYNs showed activation of MFs linked to enzyme, protein and nucleic-acid binding, whereas SYNr activated structural molecule activity and growth factor activity (Supplementary file 4, Figure S2). For the SYNCH groups in F3, SYNCHs activated BPs related to protein and metabolic processes, and both SYNCHs and SYNCHr showed suppression of detoxification and response to toxic substances; at the MF level they shared decreased structural molecule, ribosomal, actin and antioxidant activities with increased ATP-dependent chaperone and heat-shock protein binding (Supplementary file 4, Figure S3). In F4, SYNCHs showed suppressed morphogenesis and DNA-binding transcription factor activity, while SYNCHr showed activation of detoxification/response to toxic substances and translation-related functions (Supplementary file 4, Figure S3). 

In F3 embryos, SYNs showed significant positive enrichment in many KEGG pathways, including metabolic ones, while SYNr exhibited positive enrichment only for ribosomal biogenesis (Supplementary file 4, Figure S4). Among the enriched pathways in SYNCH groups in F3, SYNCHs was negatively enriched for e.g. oxidative phosphorylation, whereas SYNCHr showed positive enrichment in pyrimidine metabolism (Supplementary file 4, Figure S4). Cytoskeleton-related pathways were consistently negatively regulated across treatment groups in F3 embryos. In F4 embryos, GSEA revealed sustained positive enrichment of ribosome-related pathways in all treatment groups, with more pronounced enrichment observed in SYNr and SYNCHr groups (Supplementary file 4, Figure S4).

### RNA-seq validation

**Fig. 3:**
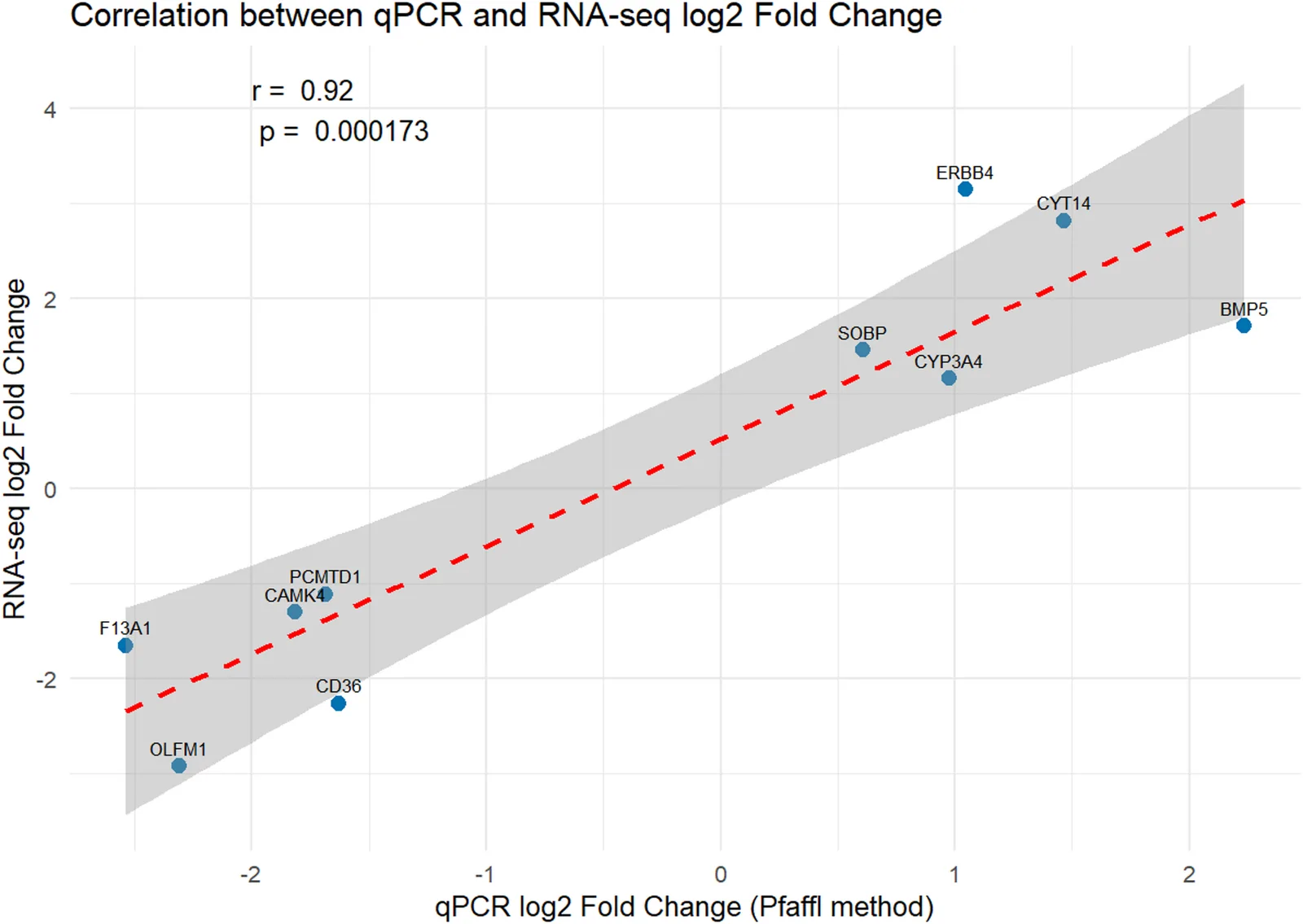
Correlation between RNA-seq and qPCR validation results. Scatter plot showing the relationship between log₂ fold change values obtained from RNA-seq and qPCR (Pfaffl method) for selected genes. A strong positive correlation was observed (Pearson’s *r* = 0.92, *p* = 0.000173), indicating high consistency between RNA-seq and qPCR expression data

The log₂ fold change values derived from qPCR showed a strong and significant positive correlation with RNA-seq data (*r* = 0.92, *p* = 0.000173), confirming the reliability and accuracy of the transcriptomic analysis (Fig. 3).

## Discussion

In this study, slow-growing Green-legged Partridgelike chicken embryos were used to investigate the effects of bioactive compounds (choline and synbiotic PS) administered *in ovo* (either as a single injection in F1 embryos or in successive F1-F3 generations) on the embryonic blood transcriptome of F3 and F4.

The *in ovo* injection method was selected for several justifiable reasons that are related to our experimental objectives. First, direct delivery of synbiotic and choline on embryonic day 12 ensured a precisely defined and reproducible embryonic exposure dose, circumventing the substantial inter-individual variability in intestinal absorption efficiency that could not be eliminated in F0 dietary supplementation approaches. Second, our *in ovo* model eliminated maternal physiological effects, including gut microbiota composition, and endocrine factors, which could introduce uncontrolled variation in the bioactive compound concentrations actually deposited into the egg. Third, the Green-legged Partridgelike chicken, as an outbred native breed, was specifically selected because outbred lines may exhibit greater susceptibility to epigenetic transmission compared to inbred lines [10]. We acknowledge that this approach does not directly follow the physiological route of nutriepigenetic exposure in free-living or commercially reared birds, where bioactive compounds are transmitted through maternal diet and yolk deposition. As such, extrapolation of our findings to naturally occurring nutriepigenetic transmission should be made with appropriate caution. However, our design was first of all serving for basic study purposes, to be able to capture the potential transgenerational effects of embryonic stimulation with bioactive compounds, in maximally controlled and repetitive conditions, and was supposed to eliminate certain “natural” factors.

The presence of DEGs in the SYNs and SYNCHs groups in F3 suggests that a single ancestral exposure may induce changes—such as epigenetic modifications affecting gene expression—that can be transmitted across generations and remain detectable two generations later as transcriptomic differences. This pattern is consistent with a potential transgenerational response. An important consideration in the interpretation of transgenerational transcriptomic data is the potential contribution of parental phenotype. Although subtle parental effects cannot be entirely excluded, the previously published phenotypic data (body weights, immune traits) from the same project [14, 15] did not indicate consistent transgenerational alterations in parental phenotype. In accordance with our results, studies across a range of species have increasingly demonstrated that environmental factors can induce epigenetic modifications that are transmitted across generations. For example, exposure of gestating F0 generation rats to vinclozolin led to disease transmission to the unexposed F3 generation [35]. In Drosophila, stress-induced heterochromatic disruption has been shown to be transmitted to multiple subsequent generations, though it gradually reverted to the normal state [36]. In line with these observations, the striking decline in the number of DEGs observed in the SYNs and SYNCHs F4 embryos in our study further supports the notion that the transcriptomic impact of ancestral exposure diminishes with increasing generational distance from the initial F1 exposure. Interestingly, in the SYN groups, *HBBA*, a gene involved in hemoglobin synthesis [9], and *BG8*, a hematopoietic BG gene associated with immune regulation [37], were the only DEGs shared between F3 and F4 in SYNs and SYNr, respectively. Notably, both exhibited reversed expression patterns: they were downregulated in F3 but upregulated in F4. This bidirectional regulation in the SYN groups may reflect compensatory mechanisms or homeostatic feedback in later generations of this lineage, as the host may attempt to restore baseline gene expression. Another explanation could be related to the dynamics of transgenerational effects, including changing expression responses, as was proposed in other studies of nutritional or environmental programming [5].

Contrary to what we hypothesized, repeated injections in the SYNr and SYNCHr groups did not result in a cumulative effect revealed in higher number of DEGs. The presence of eleven shared DEGs between SYNs and SYNr (representing approximately 8% of their combined unique DEGs: 11/(102 + 42−11)) and five between SYNCHs and SYNCHr groups in the F3 generation (approximately 8%: 5/(30 + 36−5)) suggests that certain gene networks respond consistently to synbiotic or choline stimulations, regardless of exposure frequency. For example, among the shared genes, *CYP3A4* is upregulated in all the groups of F3 embryos compared to control. This gene is involved in xenobiotic metabolism and has been linked to environmental and dietary exposures, and its persistent regulation across treatments indicates a potential core metabolic response [38]. *HIST1H4D*, a histone gene, and *COX14*, involved in mitochondrial function, suggest modulation of chromatin dynamics and cellular energy metabolism, key processes often targeted by epigenetic alterations [39, 40]. Among the shared DEGs between SYNCHs and SYNCHr, *BMP5* stands out for its established role in developmental pathways, including skeletal and neural development, and is important in regulating embryogenesis, skeletal development, and the maintenance of adult-tissue homeostasis [41, 42]. It can be suggested that specific transcriptional memory could persist in embryonic blood in response to choline and synbiotic stimulation.

The PCA results provide visual corroboration of the transcriptomic patterns identified through differential expression analysis. The separation between control and treatment groups in F3 (PC1: 29–36%) was detectable and consistent with the treatment- associated transcriptional differences identified by DEG analysis. However, the relatively modest separation and within-group dispersion may partly reflect the outbred nature of the Green-legged Partridgelike breed, which introduces individual genetic variation and may reduce statistical power to detect treatment-associated transcriptomic signals. In F4, group-level clustering became less coherent, with greater within-group variance and treatment-control separation often driven by individual samples rather than consistent group-wide patterns. This observation is consistent with the markedly reduced number of DEGs identified in the F4 generation.

Our GSEA suggested that prenatal synbiotic alone or in combination with choline modulated distinct processes and function categories. Among these, key metabolic processes and protein-related processes, including synthesis, folding, maturation, and chaperone activity, were commonly enriched in the embryonic blood of F3 and F4 embryos. Such changes may align with the reported effects of synbiotics and choline in literature. Choline is an essential nutrient involved in biosynthesis of phospholipids, neurotransmitters, and one-carbon metabolism [43]. It acts as an important methyl donor, a precursor for membrane formation, and is necessary for acetylcholine biosynthesis [44]. Choline is essential for phospholipid synthesis, which preserves cell membrane integrity [45]. Additionally, synbiotic supplementation modulates functional metabolic pathways in the intestinal microbiota, impacting host metabolism [46]. The gut microbiota significantly influences intestinal lipid and lipoprotein metabolism, affecting systemic metabolic health [47]. Microbial metabolites such as short-chain fatty acids (SCFAs) play key roles in regulating energy intake, energy harvesting, glucose and lipid metabolism, adipogenesis, immune responses, and the pathophysiology of obesity and related metabolic disorders [47]. Both animal and human studies support a strong relationship between gut microbiota composition, SCFA production, and the development or prevention of metabolic disorders [47]. The gut microbiome influences protein synthesis, cellular homeostasis, and stress response pathways [48]. Synbiotics may regulate heat shock proteins via gut microbiota interactions, enhancing mucosal immunity and stress resilience [49].

Gonadal tissues, from the same model, exhibited more persistent and tissue-specific transcriptomic signatures [19], compared to embryonic blood. This likely reflects both the transient and dynamic nature of embryonic blood during development and the cellular heterogeneity present at the sampling stage, when circulating hematopoietic cells coexist with PGCs migrating to the gonads [50]. Such compositional heterogeneity within the tissue may dilute or obscure stable transmission signals and can be considered a limitation in this study. Embryonic blood at HH-stages 14–16 comprises a heterogeneous mixture of nucleated erythrocytes, hematopoietic progenitors at varying maturation states, and circulating PGCs, meaning that observed transcriptomic differences may partly reflect shifts in cell-type proportions rather than within-cell transcriptional changes. This compositional variability could drive signals that bulk RNA sequencing cannot distinguish from genuine transcriptional regulation, potentially introducing false-positive associations. This caveat is particularly relevant when drawing mechanistic conclusions from pathway enrichment analyses. Future studies employing single-cell RNA sequencing would be valuable in disentangling these contributions and clarifying the true cellular origin of the signals reported here. Moreover, the rapid turnover and short lifespan of blood cells make them less likely to preserve early transcriptomic or epigenetic modifications, whereas long-lived or self-renewing cell populations are more capable of retaining such changes and preserve transcriptomic memory [51]. In contrast, adult tissues may represent more differentiated and stable niches that are continuously shaped by microbial, metabolic, and immune interactions [1]. It is also well established that germline-derived epigenetic alterations often manifest in a tissue-specific manner, with different somatic lineages displaying distinct transcriptomic or epigenetic outcomes [52]. Consequently, embryonic blood may carry only subtle or transient signatures that are less representative of stable inherited marks compared with adult gonadal tissue. Taken together, these factors may suggest that selecting homogenic tissue compartments may better capture the lasting, tissue-specific consequences of early nutritional and microbial programming.

## Limitation

Although our experimental design followed multiple generations and included both single- and repeated-treatment lineages, we did not directly assess epigenetic modifications such as DNA methylation, histone marks, or non-coding RNA expression. Therefore, we cannot conclusively attribute observed transcriptomic differences to stable epigenetic inheritance mechanisms. However, due to the fact that in each generation all groups were compared to the control, raised under the same housing, feed, and environmental conditions, we can deduce that the observed effect in embryonic blood can be a direct or indirect response to the administration of PS alone or with choline to eggs containing F1 embryos. Our findings should therefore be interpreted as exploratory evidence of potential multigenerational transcriptomic effects, requiring validation with phenotypic endpoints and additional molecular analyses in future work. Additionally, although the experimental model spans multiple generations, transcriptomic data were not collected from F1 and F2 embryos. This limits our ability to track the temporal progression or persistence of gene expression changes across generations. Without these intermediate datasets, it is difficult to determine whether observed patterns in F3 and F4 represent gradual changes, stable transmission, or re-emergence of gene expression shifts.

## Conclusion

This study revealed distinct transcriptomic profiles in two successive generations following *in ovo* administration of the PS synbiotic, either alone or combined with choline. Transcriptomic changes in embryonic blood were particularly pronounced in the F3 generation of Green-legged Partridgelike chickens following a single injection in F1 embryos, suggesting potential transgenerational effects of the intervention in SYNs and SYNCHs groups. The reduced transcriptomic alterations observed in the subsequent F4 generation may indicate that the intervention’s impact is strongest in earlier generations and gradually diminishes over time.

## Supplementary Information

Supplementary Material 1.

## Data Availability

The datasets supporting the conclusions of this article are available in the National Center for Biotechnology Information (NCBI) repository, https://www.ncbi.nlm.nih.gov/sra/PRJNA1358547.
