# Supplementary material for: Transcriptomic changes in embryonic blood of subsequent generations following in ovo stimulation with nutriepigenetic factors
Source: BMC Genomics. 2026 Jun 12;27:696. doi: 10.1186/s12864-026-12983-6 (PMC13495389; doi:10.1186/s12864-026-12983-6)
Supplement: Supplementary file 1 [file 12864_2026_12983_MOESM1_ESM.zip › 12864_2026_12983_MOESM1_ESM/SupplemantaryFiles/Supplementary file 4.pdf]

Table S1. The number of enriched KEGG pathways and GO terms by GSEA in chicken embryonic blood in F3 and F4 generations.

		GSEA							
		KEGG		GO: BP		GO: CC		GO: MF	
generation	comparison	Up	Down	Up	Down	Up	Down	Up	Down
F3	SYNs vs. C	15	5	12	7	0	12	12	33
	SYNr vs. C	1	1	15	5	4	4	3	1
	SYNCHs vs. C	1	4	8	3	2	9	4	5
	SYNCHr vs. C	1	2	5	37	0	7	5	23
F4	SYNs vs. C	5	2	0	0	6	0	5	2
	SYNr vs. C	7	17	0	0	0	5	0	3
	SYNCHs vs. C	1	7	0	79	0	7	0	17
	SYNCHr vs. C	24	1	59	6	31	0	30	1

C: control group; SYNs: group that received a single injection of synbiotic PoultryStar® (PS) in F1 embryos; SYNr: group that received repeated PS synbiotic injections (F1-F3); SYNCHs: group that received a single injection of synbiotic PS combined with choline in F1 embryos; and SYNCHr: group that received repeated injections of synbiotic PS combined with choline (F1-F3). GSEA: gene set enrichment analysis; KEGG: Kyoto Encyclopedia of Genes and Genomes; GO: Gene Ontology; BP: biological process; CC: cellular component; MF: molecular function.

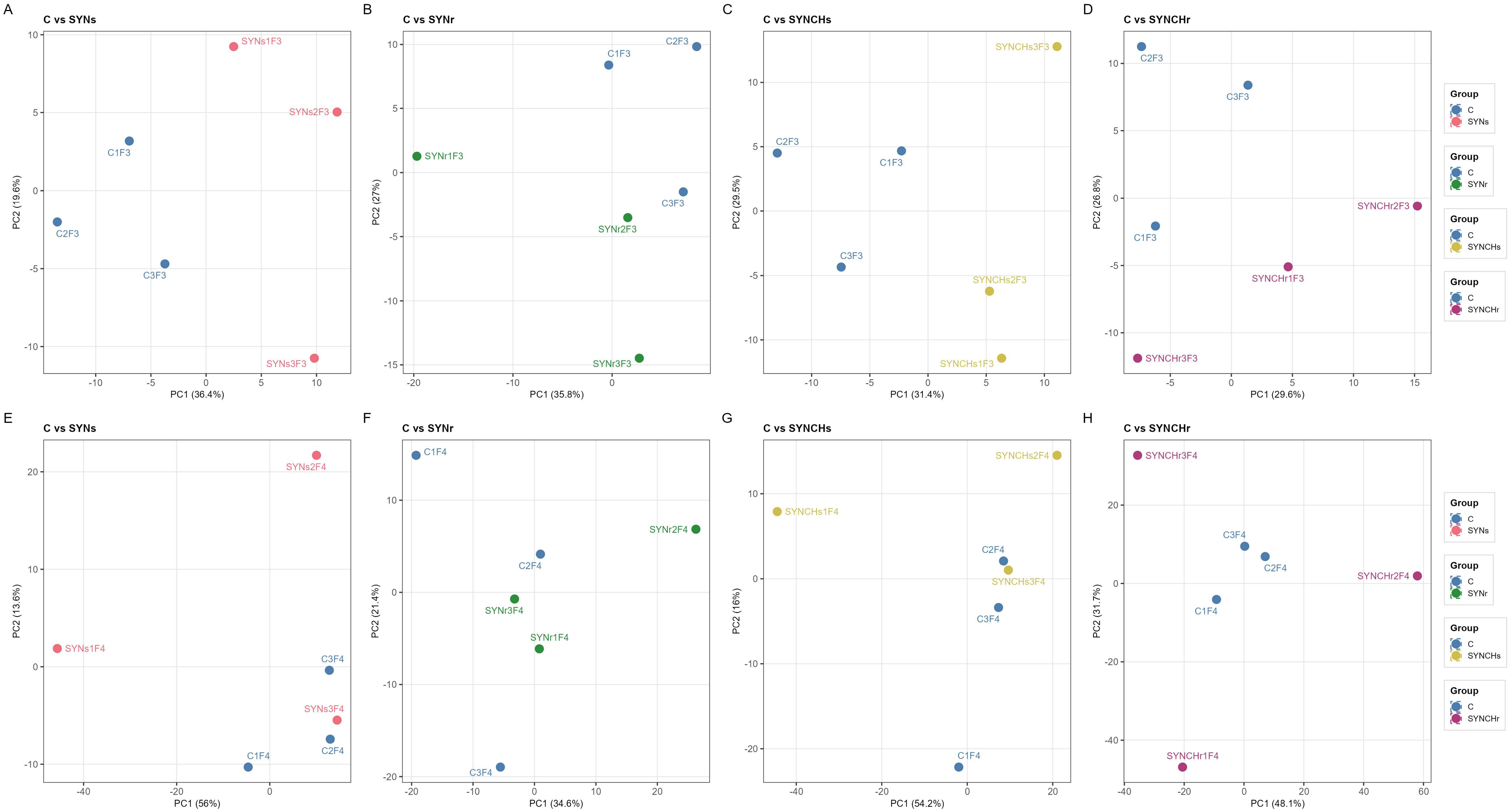

Each panel shows Control (C) vs one treatment subgroup. Dashed ellipses = 95% confidence regions. Points = individual biological replicates (n = 3). VST = variance-stabilising transformation. Green-legged Partridgelike chickens (outbred line).

Figure S1. Principal component analysis (PCA) of VST-normalised RNA-seq counts from embryonic blood across two generations. Each panel compares the Control group (C, blue) against one treatment subgroup: SYNs — single F1 synbiotic injection (A, E); SYNr — repeated F1-F3 synbiotic injections (B, F); SYNCHs — single F1 synbiotic + choline injection (C, G); SYNCHr — repeated F1-F3 synbiotic + choline injections (D, H). Upper panels (A–D) represent the F3 generation; lower panels (E–H) represent the F4 generation. Points represent individual biological replicates (n= 3). PC1 and PC2 values indicate the percentage of total transcriptomic variance explained. Analysis was performed on variance-stabilizing transformation (VST)-normalized count data using DESeq2. Green-legged Partridgelike chickens (outbred line).

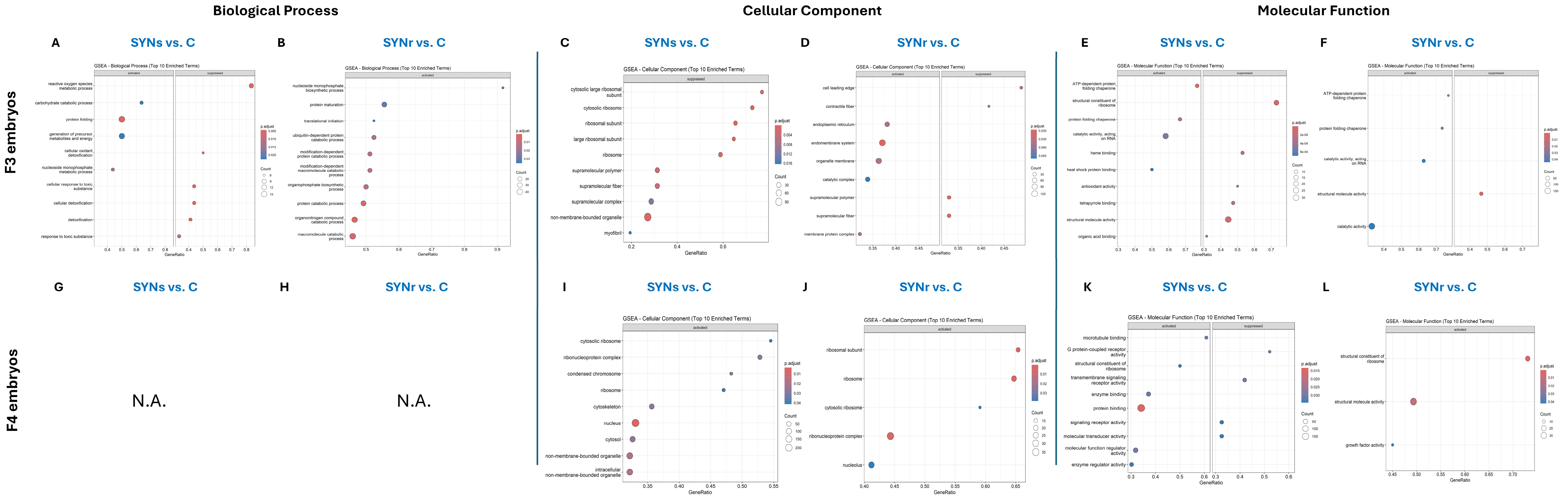

Figure S2. Enriched GO terms in single and repeatedly synbiotic injected groups by GSEA in chicken embryonic blood in F3 and F4 generation. C: control group; SYNs: group that received a single injection of synbiotic PoultryStar® (PS) in F1 embryos; SYNr: group that received repeated synbiotic PS injections (F1-F3); GSEA: gene set enrichment analysis.

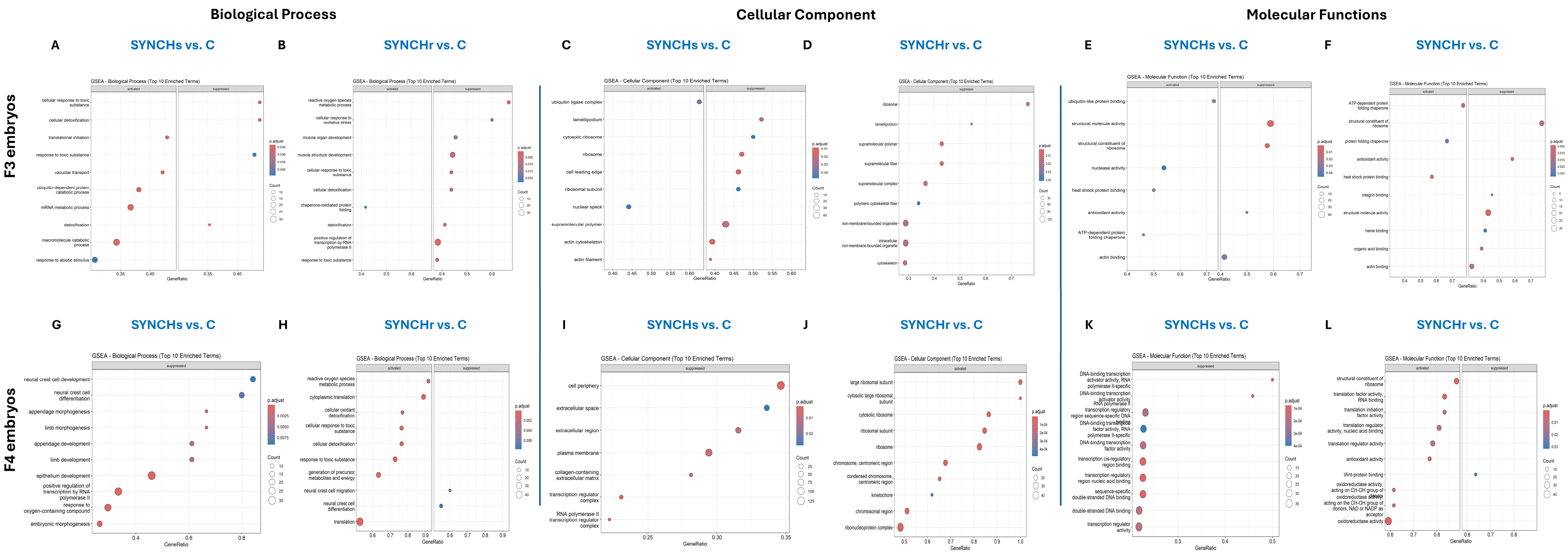

Figure S3. Enriched GO terms in single and repeatedly synbiotic + choline injected groups by GSEA in chicken embryonic blood in F3 and F4 generation. C: control group; SYNCHs: group that received a single injection of synbiotic PoultryStar® (PS) and choline in F1 embryos; SYNCHr: group that received repeated injections of synbiotic PS and choline (F1-F3). GSEA: gene set enrichment analysis.

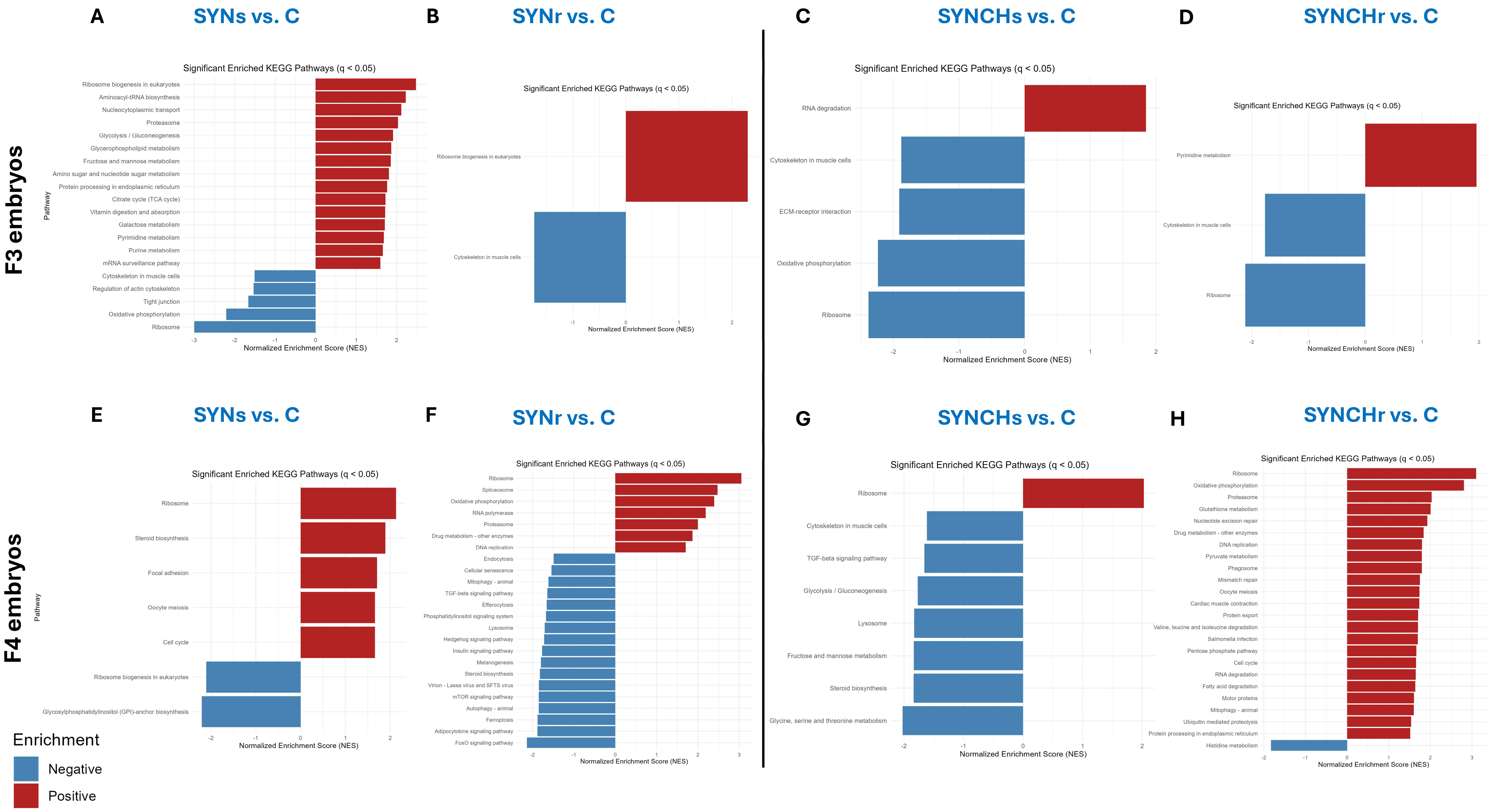

Figure S4. Enriched KEGG pathways by GSEA. (A–H) Bar plots showing the enriched KEGG pathways in SYN and SYNCH groups. Enrichment is shown for SYN groups in F3 (A, B) and F4 embryos (E, F) and for SYNCH groups in F3 (C, D) and F4 embryos (G, H). Each bar represents a pathway, with bar length corresponding to the number of enriched genes. C: control group; SYNs: group that received a single injection of synbiotic PoultryStar® (PS) in F1 embryos; SYNr: group that received repeated synbiotic PS injections (F1-F3); SYNCHs: group that received a single injection of synbiotic PS combined with choline in F1 embryos; and SYNCHr: group that received repeated injections of synbiotic PS combined with choline (F1-F3). KEGG: Kyoto Encyclopedia of Genes and Genomes; GSEA: gene set enrichment analysis
